# Possible immunoglobulin-E-dependent sugammadex-induced anaphylaxis caused by an epitope other than γ-cyclodextrin: a case report

**DOI:** 10.1186/s13256-021-02894-3

**Published:** 2021-06-05

**Authors:** Tatsuo Horiuchi, Tomonori Takazawa, Shinya Sakamoto, Masaki Orihara, Akihiko Yokohama, Mutsumi Uchiyama, Shigeru Saito

**Affiliations:** 1 Department of Anesthesiology, Gunma University Graduate School of Medicine, Maebashi, Japan; 2 Intensive Care Unit, Gunma University Hospital, 3-39-15 Showa-machi, Maebashi, Gunma 371-8511 Japan; 3 Department of Anesthesiology, Saitama Cancer Center, Ina, Japan; 4 Division of Blood Transfusion Service, Gunma University Hospital, Maebashi, Japan

**Keywords:** Sugammadex, Anaphylaxis, Basophil activation test, Phosphoinositide 3-kinase, Wortmannin

## Abstract

**Background:**

Sugammadex is a synthetic γ-cyclodextrin derivative designed to selectively bind to steroidal neuromuscular blocking agents and reverse their effects. Although many cases of sugammadex-induced anaphylaxis have been reported, few studies have investigated the underlying mechanism.

**Case presentation:**

A 55-year-old Japanese man underwent a laryngectomy under general anesthesia. One month before laryngectomy, he had undergone laryngoscopy under general anesthesia and received sugammadex administration without causing hypersensitivity. He had no history of allergies. The operation was finished without complications. Shortly after sugammadex administration, his blood pressure dropped to approximately 70 mmHg, and his heart rate increased to 110 beats/minute with systemic erythema. Suspecting anaphylaxis, he was treated with the intravenous injection of phenylephrine, d-chlorpheniramine, and hydrocortisone. After these treatments, his cardiovascular condition stabilized. Eight months after the event, skin prick tests and intradermal tests with all agents used during general anesthesia were performed. Intradermal tests showed positive results only for sugammadex. Subsequently, basophil activation tests with CD203c were performed using sugammadex, γ-cyclodextrin, and positive controls (anti-immunoglobulin-E and formyl-methionyl-leucyl-phenylalanine). In addition to both controls, sugammadex, but not γ-cyclodextrin, induced significant upregulation of CD203c expression. We performed additional basophil activation tests with wortmannin, an inhibitor of phosphoinositide 3-kinase, to investigate the mechanism underlying sugammadex-induced basophil activation. The inhibitory effect of wortmannin on basophil activation due to sugammadex was similar to that of anti-immunoglobulin-E, suggesting an immunoglobulin-E-dependent mechanism. Although the patient showed no hypersensitivity after the first exposure of sugammadex, anaphylaxis appeared after the second administration. Because most cases of sugammadex-induced anaphylaxis reportedly appeared after first administration, this seems to be a rare case.

**Conclusions:**

In the present case, sugammadex-induced anaphylaxis might have occurred through an immunoglobulin-E-dependent mechanism and not involve γ-cyclodextrin as an epitope. Physicians should pay attention to the occurrence of sugammadex-induced anaphylaxis even when the patient has a history of safe administration of sugammadex.

## Background

Sugammadex is a synthetic γ-cyclodextrin derivative that is designed to selectively bind to steroidal neuromuscular blocking agents (NMBAs) and reverse their effects. After the launch of sugammadex, however, its use has rapidly expanded because it provides quicker and more reliable antagonism of NMBAs [1]. With increasing use, many cases of sugammadex-induced anaphylaxis have been reported, suggesting that sugammadex is the leading causative agent of perioperative anaphylaxis [2, 3]. Not surprisingly, researchers are interested in the mechanisms underlying sugammadex-induced anaphylaxis. Although various hypotheses have been put forward, they have not been clarified thus far [4–6]. Here, we report a case of sugammadex-induced anaphylaxis in which we utilized the basophil activation test (BAT) to investigate the underlying mechanisms.

## Case presentation

A 55-year-old Japanese man (height, 170 cm; body weight, 76 kg) with glottis carcinoma was scheduled for laryngectomy. Written informed consent for publication was obtained from him. One month before laryngectomy, he had undergone laryngoscopy under general anesthesia and received sugammadex administration without causing hypersensitivity. He had no histories of allergies. Anesthesia was induced with 100 mg propofol and 50 mg rocuronium. At the same time, continuous administration of remifentanil was started at the rate of 0.3 μg/kg/minute. Anesthesia was maintained with 1.5% sevoflurane, 0.05–0.15 μg/kg/minute remifentanil, and 25 mg/hour rocuronium. All anesthetic injections were discontinued after the end of uneventful surgery. Two minutes after intravenous administration of 200 mg sugammadex, his spontaneous breathing was restored, and he was weaned off the ventilator. Another minute later, his blood pressure fell to 70 mmHg, and his heart rate increased to 110 beats/minute with systemic erythema. Anaphylaxis was suspected because of these cardiovascular and cutaneous symptoms, although no respiratory symptoms appeared. He was treated with intravenous injection of 0.2 mg phenylephrine, 5 mg d-chlorpheniramine, and 300 mg hydrocortisone. His cardiovascular condition stabilized after these treatments, and he was transferred to the high care unit.

Eight months after the event, skin tests were scheduled to identify the cause of anaphylaxis. Skin prick tests and intradermal tests with all drugs administered for general anesthesia were performed. Sugammadex (Bridion; MSD, Tokyo, Japan) showed positive results in intradermal tests, whereas all other agents were negative (Table 1).

**Table 1:** Results of skin tests

Drug	SPT		IDT			
	Concentration of the stock solution (mg/ml)	Results	Concentration of the stock solution (mg/ml)	Results	Wheal (mm)	Flare (mm)
Saline	9	−	9	−		
Histamine	10	+	0.01	+	15	40
Sugammadex	100	−	10	+	12	20
Propofol	10	−	1	−		
Rocuronium	10	−	0.1	−		
Remifentanil	0.05	−	0.005	−		
ABPC/SBT	20/10	−	2/1	−		
Fentanyl	0.05	−	0.005	−		
Flurbiprofen	10	−	1	−		
Acetaminophen	10	−	1	−		

Saline and histamine were used as negative and positive controls, respectively. Skin prick test (SPT) was started with a 100-fold dilution of stock solution, and a 10-fold dilution and stock solution were inspected. However, none of the drugs except histamine showed a positive result. Subsequently, intradermal tests (IDTs) were performed. After confirming a positive reaction for histamine and a negative reaction for saline, sugammadex showed a positive reaction at a 100-fold dilution of the stock solution, that is, 0.1 mg/ml. Other tested agents did not result in a positive reaction with a 100-fold dilution of the stock solution. *ABPC/SBT* ampicillin/sulbactam

**Fig. 1:**
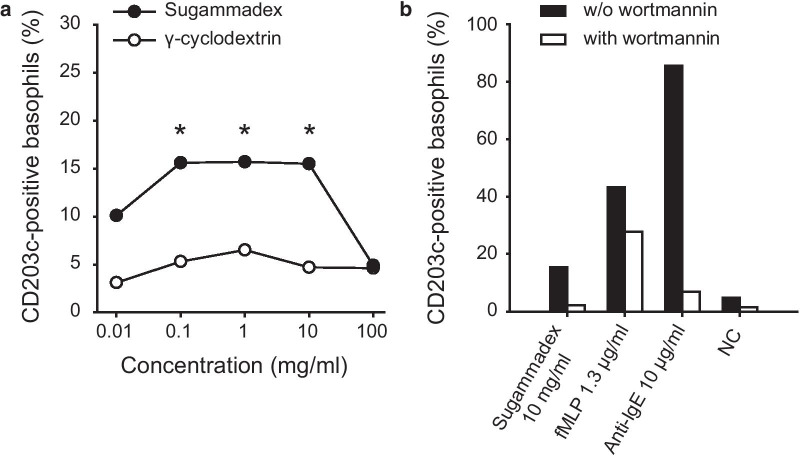
Results of basophil activation tests. **a** Sugammadex, but not γ-cyclodextrin, induced CD203c upregulation in the sugammadex-allergic patient. A maximum of 5% CD203c-positive basophils after stimulation with the buffer solution (negative control) was accepted. Asterisks indicate that the CD203c positivity rate is above the threshold. Thresholds calculated by our previous study were used to determine positivity for sugammadex [7]. **b** Upregulation of CD203c expression with and without wortmannin was compared. Wortmannin significantly suppressed sugammadex- and anti-IgE-induced CD203c upregulation. Conversely, suppression of CD203c upregulation by fMLP was limited. *NC* negative control.

Subsequently, we collected blood samples to perform BATs. The methods for conducting BATs are detailed elsewhere [7]. Briefly, the Allergenicity Kit (Beckman Coulter, Fullerton, CA, USA) was used to quantify basophil CD203c expression, a marker for basophil activation, according to the manufacturer’s instructions. Blood samples were incubated with serial dilutions of sugammadex. γ-Cyclodextrin (Wako, Osaka, Japan), which is the main structure of sugammadex and is therefore considered one of the candidate epitopes, was also tested [2, 8]. Anti-immunoglobulin E (IgE) antibody (Beckman Coulter) and formyl-methionyl-leucyl-phenylalanine (fMLP; Sigma-Aldrich, St. Louis, MO, USA) were used as positive controls. Unlike anti-IgE, fMLP activates basophils through an IgE-independent pathway [9]. Basophils in each sample were then analyzed using a flow cytometer (FACS Canto II; Beckton Dickinson Japan, Tokyo, Japan). In addition to both controls, sugammadex, but not γ-cyclodextrin, induced significant upregulation of CD203c expression (Fig. 1a).

To investigate the mechanism underlying sugammadex-induced basophil activation, we analyzed the inhibitory effect of wortmannin (Abcam, Cambridge, UK), which acts as a specific inhibitor of phosphatidylinositol 3-kinase (PI3-K) [9]. We intended to use wortmannin to differentiate between IgE-dependent and IgE-independent basophil activation. Sugammadex-induced CD203c expression on basophils was almost completely inhibited by pretreatment with wortmannin in a similar manner to anti-IgE exposure. Conversely, the effect of wortmannin on fMLP-induced CD203c expression was very limited (Fig. 1b).

## Discussion and conclusions

Although the patient showed no hypersensitivity after the first exposure of sugammadex, anaphylaxis appeared after the second administration. Because most cases of sugammadex-induced anaphylaxis reportedly appeared after first administration of sugammadex, this seems to be a rare case [2]. In the current case, sensitization might have been established after the first administration of sugammadex by production of specific IgE antibody against sugammadex.

Measurement of specific IgE antibody is an easy and reliable way to confirm the involvement of IgE in the development of anaphylaxis. At present, however, there is no commercial tool available for measurement of specific IgE antibody against sugammadex. Alternatively, BAT with wortmannin is available to investigate the involvement of IgE [10]. Because PI3-K is an essential enzyme in the IgE-mediated pathway in basophil activation, suppression of basophil activation by wortmannin suggests IgE intervention. Indeed, wortmannin almost completely suppressed basophil activation by sugammadex, suggesting an IgE-dependent mechanism of sugammadex-induced basophil activation.

Several candidates for the epitope of sugammadex have been proposed thus far. Hotta et al. previously suggested by skin test that γ-cyclodextrin might be an epitope of sugammadex [4]. In contrast, we recently performed skin tests with γ-cyclodextrin on a patient with anaphylaxis caused by sugammadex but did not observe a positive reaction [6]. Because the results of the current case again suggested that γ-cyclodextrin is not necessarily an epitope, further studies are needed to clarify this issue.

Recently, an increasing number of patients with positive skin tests for sugammadex-rocuronium complex but negative for sugammadex has been reported [5, 8]. There have been many cases in which sugammadex alone showed positive results in skin tests, as in this case; thus, multiple mechanisms of sugammadex-induced anaphylaxis are considered [6].

In conclusion, we demonstrated that sugammadex-induced anaphylaxis might have occurred through an IgE-dependent mechanism and not involved γ-cyclodextrin as an epitope. Physicians should pay attention to the occurrence of sugammadex-induced anaphylaxis even when the patient has a history of safe administration of sugammadex.

## Data Availability

Data relevant to this case report are not available for public access because of patient privacy concerns but are available from the corresponding author on reasonable request.

## Abbreviations

NMBA: Neuromuscular blocking agent
BAT: Basophil activation test
IgE: Immunoglobulin E
fMLP: Formyl-methionyl-leucyl-phenylalanine
PI3-K: Phosphatidylinositol 3-kinase
SPT: Skin prick test
IDT: Intradermal test
ABPC/SBT: Ampicillin/sulbactam
